# Does external reference pricing deliver what it promises? Evidence on its impact at national level

**DOI:** 10.1007/s10198-019-01116-4

**Published:** 2019-10-03

**Authors:** Panos Kanavos, Anna-Maria Fontrier, Jennifer Gill, Olina Efthymiadou

**Affiliations:** Department of Health Policy, LSE Health – Medical Technology Research Group, Cowdray House, London School of Economics and Political Science, Houghton Street, London, WC2A 2AE UK

**Keywords:** External reference pricing, Pharmaceutical pricing, Pharmaceutical policy, Regulation of pharmaceuticals, Systematic review, Expert consultation, I, I1, I10, I11, I18

## Abstract

**Background:**

External reference pricing (ERP) is widely used to regulate pharmaceutical prices and help determine reimbursement. Its implementation varies substantially across countries, making it difficult to study and understand its impact on key policy objectives.

**Objectives:**

To assess the evidence on ERP in different settings and its impact on key health policy objectives, notably, cost-containment, pharmaceutical price levels, drug use, equity, efficiency, availability, affordability and industrial policy; and second, to critically assess the quality of evidence on ERP.

**Methods:**

Primary and secondary data collection through a survey of leading experts and a systematic literature review, respectively, over the 2000–2017 period.

**Results:**

Forty five studies were included in the systematic review (January 2000–December 2016). Primary evidence was gathered via survey distribution to experts in 21 countries (January–July 2017). ERP contributes to cost-containment, but this is a short-term effect highly dependent on the way ERP is designed and implemented. Low prices, as a result of ERP, can undermine the availability of medicines and lead to launch delays or product withdrawals. Downward price convergence can hamper investment in innovation. ERP does not seem to promote efficiency in achieving health system goals. As evidence is weak, results need to be interpreted with caution.

**Conclusions:**

ERP has not regulated prices efficiently and has unintended consequences that reduce the benefits arising from it. If ERP is carefully designed with minimal price revisions, prudent selection of basket size and countries, and consideration of transaction prices, it could be a more effective mechanism enhancing welfare, equitable access to medicines within countries and help promote industry innovation.

## Introduction

External Reference Pricing (ERP) is used widely across the globe to inform decisions on pricing and coverage of pharmaceuticals and is used either as the dominant method to explicitly set prices or as one of the criteria to inform pricing and reimbursement decisions [1–7]. In general, ERP operates on the basis of identifying prices from a basket of reference countries, the selection of which is based on four main criteria: (i) geographic proximity to the benchmark country; (ii) comparable GDP levels; (iii) similar socioeconomic conditions; and (iv) ad hoc considerations in the benchmark country, such as ‘desirable’ price levels [7, 8]. In the majority of cases, ex-factory prices are used to inform pricing decisions and pricing authorities rely mostly on list prices rather than transaction prices to do so [1–7]. The method used to calculate the reference price usually differs across countries; often, the lowest in the basket is used but it is not uncommon to use the average or the median [2, 3, 5–9]. The number of countries considered in the basket as well as the frequency of price revisions also varies across countries. As a result, ERP systems vary substantially in the way they are implemented and, consequently, the impact of ERP is difficult to study compared to other pricing methodologies. Additionally, the way ERP is implemented in a particular setting is crucial due to the path-dependant nature of ERP, in that the features of ERP influence the size and extent of its impact in that setting. Hence, the prudent implementation of ERP can be translated in the attainment of the overarching policy objectives set within countries, such as availability, affordability and equity.

By reviewing and synthesising evidence from primary and secondary sources, the objective of this paper is to gain a clearer understanding of the impact ERP has on important health system goals such as cost containment, price levels, pharmaceutical consumption, availability and affordability, efficiency, equity and industrial policy, as well as to determine which factors might affect these goals positively or negatively. An additional objective is to provide an assessment of the quality of the available evidence, its robustness and, by implication, its suitability to inform policy.

Our study differs from other similar studies in this area [6] in a number of ways. First, we combine secondary evidence with primary data collection such that the latter addresses gaps in the former. Second, we capture primary evidence on the impact of ERP in 21 countries in Europe, the Middle East, the Russian Federation, Brazil and South Africa. Third, in comparison to previous studies, which tend to examine a combination of national and international effects of ERP, our study provides an in-depth analysis of the performance of ERP against policy objectives countries have at national level only. Finally, as there is a lack of empirical studies with clear methodological design, lack of long-term evidence and scarce evidence on the reasons why ERP impact varies across countries, we critically assess the quality of evidence found in the literature and study the magnitude of ERP effects over time. To our knowledge, no other study provides an up-to-date review and analysis of the impact of ERP on health system goals within countries, appraising the quality of evidence, or by drawing upon the breadth of country settings that we do.

## Conceptual background

Decision makers resort to regulating the prices of new and in-patent pharmaceutical products because in-patent pharmaceutical markets are characterised by a significant degree of monopoly or oligopoly. Upon entry and as a ‘first-in-class’, a new pharmaceutical product commands monopoly power, while entry of similar, but slightly distinct, products in the same therapeutic category (also known as me-too products), in principle enables a degree of competition depending on whether therapeutic options in the same indication are considered to be perfect or imperfect substitutes. Still, the number of entrants in individual therapeutic indications is increasingly becoming smaller, as indications are becoming narrower, pointing to oligopolistic markets, where the options are slightly differentiated in relation to their therapeutic, clinical or safety features. As such, therefore, in-patent pharmaceutical markets are characterised by monopolistic competition. By leveraging intellectual property rights and the protection they offer, innovators have an incentive to behave as monopolists and, as a result, enter the market and price at monopoly level.

Regulators, on the other hand, have an incentive to curb monopoly power. Different strategies can be adopted in this context, not all of which are mutually exclusive: first, they can assess the value of new products in a therapeutic indication through comparative clinical benefit assessment or clinical and cost-effectiveness analysis or, even, an enhanced clinical and cost-effectiveness analysis, which involves the inclusion of additional dimensions of value beyond incremental costs and effects; through that process, the price and the expected utilisation of a new pharmaceutical product are going to be determined. Second, they can adopt a costing methodology, whereby innovators are required to submit evidence on their cost structure, including R&D costs, and allowing a return on investment, which is fixed; this is known as a cost-plus approach and has been used in the past to determine prices of pharmaceuticals in many settings. Third, they can control the return on sales (profit) or the rate of return on capital employed instead of controlling prices, which is a method that addresses profitability or return on capital, rather than price control. And, fourth, they can implement explicit price regulation and one form of that is comparative in nature, whereby regulators resort to borrowing prices from other settings, which is known as external reference pricing (ERP).

The popularity of ERP over the past quarter of a century lies in its simplicity, relative ease of adaptation and flexible design features, which can be used to guide and meet pharmaceutical policy objectives. Key design features of ERP include, first, the selection of reference countries, which can be adapted to guide price diminution; second, flexibility in the selection of the reference price to inform price setting, where the focus can shift from the average or median in the basket, to the average in the lowest decile of reference countries, or, simply, the lowest in the basket; third, the frequency of re-pricing or price revisions, which enables the inclusion of more countries from the basket with the aim to cause further price reductions; fourth, the use of exchange rates as a means of guiding price reductions, particularly, in circumstances where significant currency movements are observed; and, fifth, the use of differential intellectual property expiry dates as a means of causing further price diminution, by taking generic prices in patent-expired ‘reference’ markets and applying these in the ‘domestic’ market.

The application of ERP and its design characteristics may contribute to price optimisation and cost containment (or macroeconomic efficiency); however, it is also important to consider the impact this practice is having on key policy objectives such as utilisation (volume), access (both in terms of availability and affordability), efficient resource allocation (as opposed to macroeconomic efficiency), and industrial policy.

## Methods

In order to study the impact of ERP at country level and how its implementation contributes to national policy objectives, evidence from both primary and secondary sources was collected. Secondary evidence was collected via a systematic literature review (SLR), which was carried out in accordance with the CRD guidelines for systematic reviews [10]. SLR evidence was validated, complemented and updated via primary data collection, carried out by surveying key experts across 21 countries, which were known to have implemented ERP either as their main method of or as a criterion for pharmaceutical price-setting.

### Systematic literature review

#### Analytical framework

To inform the SLR, an analytical framework was created to critically assess the impact of ERP within countries. In that context, other frameworks for health systems and pharmaceutical sector policy implementation were identified and enhanced for this study. The WHO [11] and the OECD [12] have argued that the impact of pricing policies should be monitored beyond the management of prices [11, 12]; ultimately, pricing policies must balance both static efficiency, namely keeping prices low relative to benefits, and dynamic efficiency, such as encouraging innovation through a variety of actions, including an active industrial policy [11–13]. However, these two types of efficiency are inter-connected as initiatives aiming to stimulate system-wide objectives, such as availability and access, require sustainable price management [11].

WHO guidelines on country pharmaceutical pricing policies [11] highlight that in order to promote the use of affordable medicines, countries should employ pharmaceutical policies that address both supply- and demand-side imperfections. Beyond the management of prices, policies should align with principles of the wider health and pharmaceutical setting within which they operate and must be tailored accordingly: they should promote equitable access and ensure affordability and their impact should be monitored not only in terms of their effect on price levels but also on the influence they have on other outcomes, such as availability of essential medicines [11]. Similarly, the OECD assesses various pharmaceutical pricing and reimbursement policies that have an impact on access to effective medical treatments. In addition, it investigates the trade-off in pharmaceutical policies between static and dynamic efficiency. By ensuring the lowest possible prices for pharmaceuticals today, there may be implications for both the availability of pharmaceuticals and the incentives for R&D, in the future [12].

Based on the above, we endeavoured to reflect both on the immediate impact of pricing policies (impact on healthcare and pharmaceutical budgets, price levels, drug use and consumption) and on performance measures of health system-wide goals (availability, affordability, equity, microeconomic efficiency and industrial policy). The features of this framework, shown in Table 1, served as our study endpoints.

**Table 1:** ERP and its impact within countries: Summary of the analytical framework. Source: The authors

System-wide health policy objectives	Definition	Issues raised
Cost containment	Examines the extent to which ERP has the capacity to reduce or contain the rate of increase in pharmaceutical spending	ERP can lead to health care system savings
		Extent of savings depends on the way ERP is implemented
Price levels	Assesses whether ERP leads or is able to secure reasonable prices for payers and healthcare systems	ERP secures low pharmaceutical prices
		Pharmaceutical prices depend on ERP design
		Pharmaceutical prices depend on market features
Drug use	Assesses whether ERP can manage excessive drug consumption	ERP impacts diffusion and use
Availability	The extent to which new pharmaceuticals are available in the market for which they are intended	ERP can lead to market withdrawal
		ERP can lead to launch delays, launch sequencing or no launch
Affordability	The extent to which pharmaceutical prices are congruent with the purchasing ability of health care systems and/or patients	ERP leads to prices in line with the purchasing ability of health care systems and/or patients
		ERP enhances the scope for affordability
Fairness/social welfare	The ability of ERP to promote equitable access to medicines	ERP has an impact on social welfare
		ERP has an impact on health system priorities
Microeconomic efficiency	The extent to which ERP promotes health system efficiency and leads to optimal resource allocation	ERP has an impact on efficient drug expenditure
		ERP is associated with a stable share of pharmaceutical expenditure on total health spend
		ERP helps contain costs while guaranteeing access to medicines
Industrial policy	Assesses whether ERP promotes and/or is consistent with the objectives of industrial policy (attracting manufacturing, R&D and/or related activities) or it acts as a barrier to attracting these	ERP impacts innovation and investment in R&D
		ERP influences manufacturing and/or R&D investment decisions
		ERP can promote innovation

#### Inclusion criteria (study endpoints, country selection, and study period)

Evidence was collected according to a set of predefined endpoints, based on the analytical framework presented in Table 1, and issues that each endpoint would address. For example, under ‘cost-containment’, the following issues were identified: (i) the ability of ERP to generate healthcare savings and (ii) the impact of the ERP design on cost containment. There were no country-specific restrictions imposed on our search to ensure that evidence from a wide geographical range was collected. The study period for inclusion of relevant published studies was from January 2000 to December 2016. The start-date coincides with the period many countries started to implement ERP.

#### Identification of evidence (data sources and search strategy)

Seven electronic databases were searched for peer-reviewed literature (Web of Science (WoS), CINAHL, EconLit, Medline, ProQuest, the Cochrane Library and Scopus) using the following keywords: “Pharmaceutical Price Regulation” OR “Pharmaceutical Regulation” OR “Cost Containment” OR “Pharmaceutical Pricing” OR “External Reference Pricing” OR “External Price Referencing” OR “International Price Comparisons” OR “International Reference Pricing” OR “International Price Referencing” AND drug OR drugs OR medicine OR medicines OR pharmaceutical OR pharmaceuticals. In addition to the peer review and grey literature, a targeted search of the WHO, the WHO Collaborating Centre for Pharmaceutical Pricing and Reimbursement Policies–Gesundheit Österreich GmbH (WHOCC–GOEG), the OECD online databases and the European Commission was carried out to ensure that no relevant reports were omitted. Relevant information was recorded and combined with the results of the systematic literature review.

#### Study selection and data extraction

The following stages were followed to select studies and extract data in adherence with the CRD guidelines: first, search results were filtered based on the relevance of the title and abstract to the topic. Evidence from grey literature was also included in the systematic review to ensure that all relevant studies were considered. Studies with relevant titles were downloaded for further examination. The main body of these texts was then assessed for relevance against the inclusion criterion: ‘mention of external reference pricing and its impact within countries’ at least in one of the selected endpoints such as price levels, affordability and access to provide a final set of potentially relevant studies. The number of documents presenting evidence on each endpoint was noted. Where one study presented evidence on more than one endpoint, this was recorded separately each time. An excel spreadsheet was used to extract the relevant information on each endpoint from the final set of studies included in this study. The spreadsheet comprised titles of studies in rows versus the endpoints in the columns, with important information from the texts being extracted. Where the search yielded studies, which were the product of a systematic literature review, these were only included in our analysis if the endpoints considered were different from those set out here, in order to gather as much information as possible whilst at the same time avoiding any possible bias.

#### Quality of evidence

As impact assessment studies in pharmaceutical policy have been found to be weak, often casting doubt on many of the conclusions drawn [14], we critically assessed the quality of evidence used in this study by appraising the methodological design of the identified studies. Studies with strong quasi-experimental designs (e.g. time series with a comparison group) and randomised controlled trials (RCTs) are considered to be well controlled compared to before–after or post-only studies, which are considered to be weak research designs, often producing unreliable impact assessments [14]. We categorised the studies into empirical and non-empirical. The former comprised any randomised and non-randomised controlled trials, studies using quasi-experimental designs and other quantitative analyses such as before-after and post-only designs. The latter included theoretical models, descriptive studies or other literature reviews.

To assess whether we could draw meaningful conclusions from our findings, we recorded both the study design and timeline of all identified studies. The type of the study design would enable us to make inferences about the robustness of the evidence and the extent to which it could provide meaningful policy recommendations. Study timelines would enable us to make inferences about short- or long-term effects. We considered all study designs, both experimental (e.g. RCT) and quasi-experimental, for the latter applying a hierarchy that distinguishes between different strengths (time series with comparator group; pre-post with a comparator group; pre-post without comparator; post only) [14]. In terms of the study timelines, we considered the likely effects of ERP on a policy variable or endpoint of 3 years or less to be “short-term” in nature. A simple vote-counting methodology was used to determine the accumulated impact of ERP on each endpoint and issues identified within each endpoint. An overall scorecard was developed, based on three dimensions: first, the direction of impact (i.e. positive or negative) that ERP was found to have on the identified endpoints and issues; second, the quality of the empirical evidence considered under individual endpoints and issues; and third, the extent to which the studied endpoints and issues have been studied explicitly in the identified literature.

### Primary data collection

Based on identified country evidence from the SLR, we conducted a follow-up primary data collection to validate, update and enhance our findings. Primary evidence was generated using a short survey, which was sent to key stakeholders in 26 countries between January and July 2017. These countries were selected in the sample as ERP was used either as a primary or supportive tool in price setting at the time of contact. The selected stakeholders were leading health and pharmaceutical policy experts in their countries and were affiliated with national competent authorities (e.g. regulatory agencies, pricing departments or reimbursement agencies), or academic and research organisations. The views of pharmaceutical industry representatives were also requested. The stakeholders were asked to provide evidence, and their personal perspective, on the impact of ERP within the countries they were responding on. The survey included six questions to provide feedback on, tailored around the endpoints and the associated issues shown in Table 1. The views of the expert responders were their own and did not represent the view of their institution. Stakeholders’ responses to the survey are referred within the text as ‘primary evidence, primary data or survey’ [15].

## Results

### Evidence generation from the systematic review and primary data collection

**Fig. 1:**
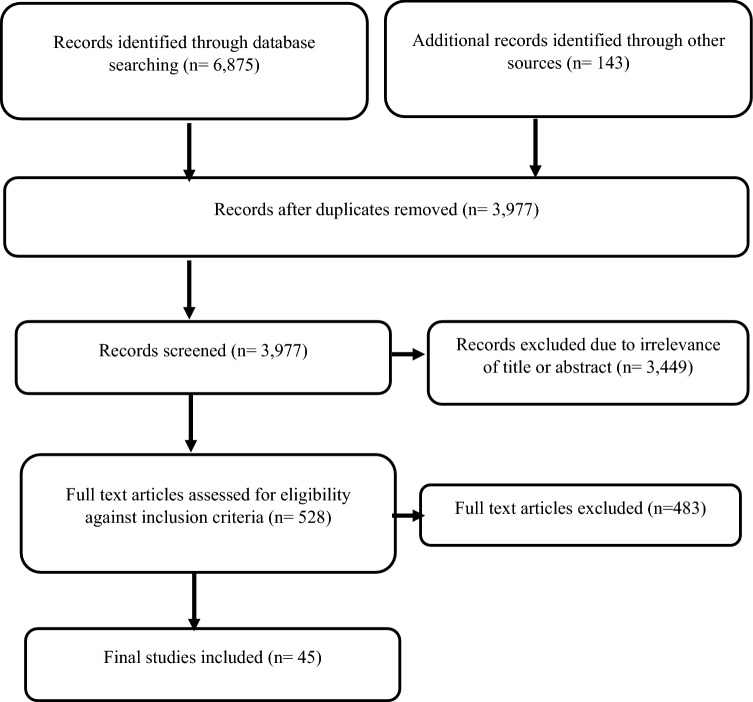
PRISMA flow diagram outlining search results for the systematic literature review. Source: The authors

Figure 1 shows the PRISMA flow diagram of the review process and the respective number of articles at each stage. The database search yielded 6875 studies. The results of the systematic literature search were then combined with the results from the targeted grey literature search. By removing duplicates using the EndNote software, 3977 studies were initially screened based on relevant titles and abstracts. From the 3977 studies, 3489 were peer reviewed and 488 were grey literature. Of the 3977 studies 3449 records were excluded due to irrelevance of title or abstract and 528 studies were downloaded and assessed for eligibility. Studies were excluded for either non-relevance to ERP or when internal reference pricing was studied. In addition, studies were excluded when only their abstracts were available and when studies were focusing on the international implications of ERP, which falls outside the scope of this review. A total of 45 studies were included in this systematic review.

Three studies included in this systematic literature review were empirical (6% of all studies considered). The majority of studies were descriptive in nature but also included theoretical models or literature reviews. When examining the impact of ERP against the included endpoints, a post-only design was used to capture the impact of ERP quantitatively.

Primary data yielded important information on the modus operandi of ERP and its impact on key endpoints. Twenty-one countries (Belgium, Brazil, Bulgaria, Egypt, Estonia, France, Germany, Greece, Hungary, Italy, Jordan, Latvia, Poland, Portugal, Qatar, Romania, The Russian Federation, Slovakia, Slovenia, South Africa and Spain) responded to the survey, whereas five countries did not return a response (Austria, Czech Republic, Portugal, Saudi Arabia and Turkey). A total of 23 key stakeholders (one per country, except for Brazil and Hungary, that had 2 respondents each) participated in the survey.

**Fig. 2:**
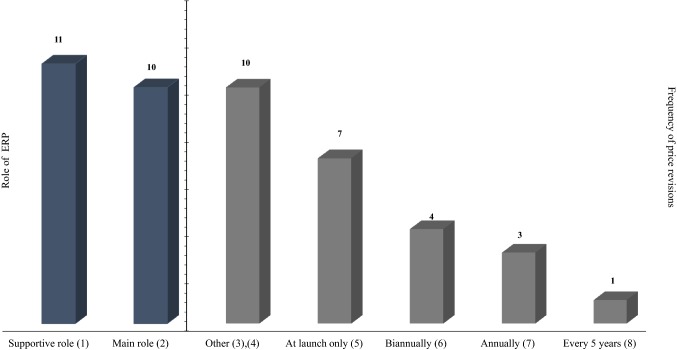
Salient features of ERP in 21 countries: role of ERP and price revision frequency*. Notes* The frequency of price revisions does not total 21 as many countries use more than one method to determine price revision frequency. For example, in Poland price revisions are taking place on an ad hoc basis and periodically at tiered intervals (every 2, 3, or 5 years). (1) Countries where ERP has a supportive role to price setting: Belgium, France, Germany, Hungary, Italy, Latvia, Poland, Spain, Brazil, The Russian Federation, Estonia. (2) Countries where ERP has a main role in price setting: Bulgaria, Greece, Portugal, Romania, Slovakia, Slovenia, Egypt, Jordan, Qatar, South Africa. (3) “Other” includes ad hoc, availability of new evidence, specific agreements, upon manufacturer’s request, etc. (4) Countries in the “other” frequency of price revisions: Estonia, Germany, Italy, Latvia, Poland, Spain, Egypt, Jordan, Russia, South Africa. (5) Countries in the “at launch only” frequency of price revisions: Belgium, Bulgaria, Germany, Hungary, Egypt, Qatar, Brazil. (6) Countries in the “biannually” frequency of price revisions: Greece, Bulgaria, Slovakia, Slovenia. (7) Countries in the “annually” frequency of price revisions: Estonia, Portugal, Romania. (8) Countries in the “every 5 years” frequency of price revisions: France. *Source:* The authors based on the literature review findings and primary data collection

**Fig. 3:**
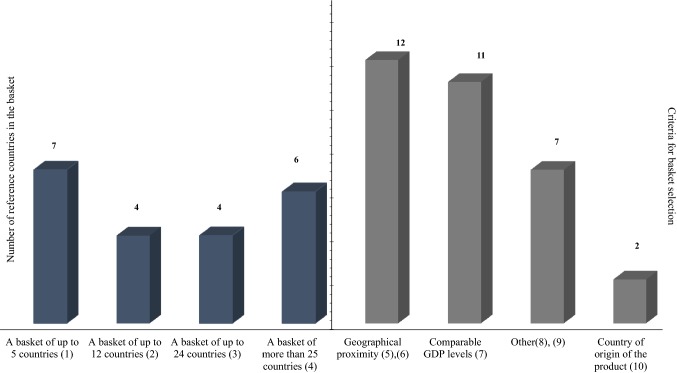
Salient features of ERP in 21 countries: Basket size and country selection criteria*. Notes *The criteria for basket selection do not total 21 as many countries use more than one method to select their basket. (1) Countries with a basket of up to 5 countries: Estonia, France, Portugal, Slovenia, Qatar, The Russian Federation, South Africa. (2) Countries with a basket of up to 12 countries: Bulgaria, Latvia, Romania, Brazil. (3) Countries with a basket of up to 24 countries: Germany, Greece, Spain, Jordan. (4) Countries with a basket of more than 25 countries: Belgium, Hungary, Italy, Poland, Slovakia, Egypt. (5) The “geographical proximity” also included European countries such as Eurozone countries. (6) Countries selecting their basket based on geographical proximity: Belgium, Estonia, France, Germany, Greece, Hungary, Latvia, Poland, Slovakia, Slovenia, Spain, and Egypt. (7) Countries selecting their basket based on comparable GDP levels: Belgium, Bulgaria, Estonia, France, Germany, Italy, Latvia, Portugal, Egypt, Brazil, and The Russian Federation. (8) “Other” includes countries where prices are available and accessible, no clear criteria, etc. (9) Countries selecting their basket using other criteria: Bulgaria, Latvia, Romania, Jordan, South Africa, Qatar, Romania. (10) Countries including in their basket the product's country of origin: Egypt, The Russian Federation. *Source:* The authors based on the literature review findings and primary data collection

The role of ERP in these countries was found to range from “supportive” to “main method” in price setting [9]. Evidence on the frequency of price revisions showed that many countries revise their reference prices based on reasons such as availability of new evidence for specific pharmaceuticals or on specific agreements made at national level; a third of the countries use ERP at launch only, while others do not have a clear structure for price revisions and price recalculations may take place, for instance, every 6 months or at manufacturers’ request [9]. The majority of the countries of interest select reference countries based on geographical proximity, while others take into consideration GDP levels. The size of the basket may vary from very small (up to five countries) to very large (more than 25 countries) [9]. These features are summarised in Figs. 2 and 3.

### Results by endpoint

In the section of the results below, we endeavoured to match the country-specific literature findings with the evidence collected from primary data. Therefore, both primary and secondary evidence is presented in alignment. A summary of all available evidence along with issues identified for each endpoint is presented in Table 2.

**Table 2:** Summary of evidence on the impact of ERP on key policy objectives. Source: The authors based on the literature review findings and primary data collection

Endpoints	Issues	Overall evidence from primary and secondary sources	Countries with primary and secondary evidence	Quantifiable impact	Short- vs. long-term effect
(1)	(2)	(3)	(4)	(5)	(6)
Cost-containment	ERP can lead to health care system savings	European countries are introducing ERP to contain costs and increase healthcare savings. The evidence on the impact of ERP on savings varies across countries [1, 12, 16, 17, 19, 20]	Slovakia, Turkey, Greece, Switzerland	Slovakia, 2012: Eur €75 m Turkey, 2007: US$900 m-US$1bn	At least in the short-term, ERP can be used as a tool to control costs, whereas in the long-term its impact on cost-savings is uncertain [1, 19, 20]
	Extent of savings depends on the way ERP is implemented	The extent of healthcare savings depends largely on the way ERP is implemented. Frequent price revisions and consideration of transaction prices could result in higher sustained savings [1, 12, 20, 21]	All EU countries and Switzerland	*‘Consideration of actual discounted prices’* *‘Frequent price revisions’*	
Price levels	ERP secures low pharmaceutical prices	There is a mixed evidence on the ability of ERP to secure low prices [4, 5, 15, 16, 19, 22–26, 28–31]	The Netherlands, Cyprus, Norway, Romania, Bulgaria, Greece, Slovakia, Moldova, China, Belgium, Brazil, Germany, Egypt, Spain, Estonia, France, Hungary, Italy, Latvia, Romania, The Russian Federation, Slovenia, South Africa, Qatar, Jordan	The Netherlands, between 2007 and 2008: 8% decrease of POM prices; Moldova, 2012: Pharmaceutical prices decreased by 3%; Bulgaria, 2014: Prices of reimbursed pharmaceuticals decreased between 4 and 75.4%; Greece, 2010: Pharmaceutical prices decreased by an average of 9.5%	A limited number of studies consider long-term evidence when studying the impact of ERP on pharmaceutical prices. Therefore, whether ERP can or cannot continue to reduce prices over time is still unclear [1–8, 12, 25, 38, 39]
	ERP as a meaningful regulation to lower pharmaceutical prices both at launch and over time	Evidence has shown that ERP reference prices (which are based on list prices in basket countries), rather than actual transaction prices in the basket countries, lead to higher pharmaceutical price levels in countries implementing ERP and limit the opportunities for these countries to benefit from the transaction prices attained in individual countries [1, 2, 4–8, 15, 40]	All EU and OECD countries	N/A	
	Pharmaceutical prices depend on ERP design	The extent of reduction in pharmaceutical prices depends largely on the design of the implemented ERP. Frequent price revisions, larger country baskets, 'wiser' basket country selection and the consideration of the average or the lowest prices in the basket when calculating the reference price, can lead to even more downward pressure on price levels [4, 6–8, 15, 21, 22, 26, 28, 30, 32, 33]	Croatia, Austria, Belgium, Cyprus, Denmark, Estonia, Germany, Iceland, Luxemburg, Poland, Greece, Latvia, Lithuania, Slovakia, Switzerland, Moldova, Hungary, Spain, Bulgaria, The Russian Federation	*‘Larger basket’* *‘Basket country selection’* *‘Frequent price revisions’* *‘Calculation of reference price based on average or lowest prices in the basket’*	
	Pharmaceutical prices depend on prevailing market features	Pharmaceutical price levels correlate with country GDP per capita and can be affected by levels of market regulation, including ERP, and any economic pressure applied in the studied country [4, 5, 15, 37–39]	Norway, The Netherlands, Finland, Austria, Belgium, Spain, Greece, Portugal, Lithuania, Qatar	*‘Lower GDP levels’* *‘Strict price regulations’* *‘Other fiscal concerns’*	
Drug use	Improved drug diffusion and use	There is mixed evidence on the impact of ERP on pharmaceutical diffusion and use. Some countries documented that ERP is not a sufficient condition for the diffusion and use of pharmaceuticals, whereas others supported that pharmaceuticals diffuse well with ERP [15]	Brazil, Egypt, France, Greece, Hungary, Latvia, Qatar, Slovakia, Spain, Bulgaria, Estonia, The Russian Federation, Italy, Jordan	N/A	There is no long-term evidence on whether ERP is a sufficient condition for improved diffusion and use of pharmaceuticals
Availability and launch delays	Market withdrawal	ERP may result in a general price decrease when one country reduces its price, suggesting that if the price generated becomes too low, manufacturers may withdraw from the market resulting in the product becoming unavailable [2, 3, 6, 15, 28, 36]	Bulgaria, Spain, Estonia, Romania, Slovakia, Poland, Greece, Latvia	Bulgaria: 200 products were withdrawn from the market in 2012	Short-term: In Bulgaria 200 products were withdrawn in 2012 Long-term: Within a 6 year period, 11 products among 7 EU countries were not launched by manufacturers in order to avoid expected low prices [6, 7, 25]
	Launch delays, launch sequencing or no launch	Companies may delay, sequence or withhold drug launches in countries with highly controlled prices at ex-factory level or in countries where price levels are considered low [1, 3–8, 15, 36]	Slovakia, Hungary, Germany, Belgium, Poland, Estonia, Spain, Romania, Bulgaria, Greece, Latvia	Belgium: systematic delay of dossier submission by companies in order to avoid the Belgian price Some companies tried to ignore the process or actively lobby for exemptions for their products; Slovakia: after a change in its country basket to include all EU Member States; Germany: had the highest availability among 15 European countries; 11 products among 7 EU countries were not launched by manufacturers in order to avoid expected low prices	
Affordability	ERP leads to pharmaceutical prices in line with the purchasing ability of healthcare systems or patients	It has been noted that countries with high absolute price levels of pharmaceuticals, have low relative price levels (pharmaceutical prices divided by GDP per capita). Nevertheless, ERP policies (and the way ERP systems are designed) encourage higher prices in LICs, directly undermining affordability of pharmaceuticals in these countries. The majority of countries though experience affordability issues at least in some therapeutic classes [1, 2, 7, 15, 23, 44, 51]	Slovakia, Belgium, Brazil, Bulgaria, Spain, France, Greece, Hungary, The Russian Federation, South Africa, Jordan, Germany, Denmark, Ireland, Italy, Poland, Romania, Egypt, Qatar, Latvia, Estonia	Germany, Denmark, Ireland and Italy, have low relative price levels (pharmaceutical prices divided by GDP per capita); Poland, Romania and Bulgaria, pay relatively more compared to their GDP per capita; In Egypt, prices are high for the local population in relation to per capita income	There is no conclusive and/or empirical evidence that ERP undermines affordability over time
	Scope for increasing affordability	If reference prices are set based on some kind of affordability index which reflects national income levels, either through an average exchange rate or PPPs, affordability in LICs could be improved [1, 2, 44]	Egypt	N/A	
Fairness/Social Welfare	ERP impacts social welfare	ERP and parallel trade had an effect on social welfare by increasing prices, therefore, undermining equitable and affordable patient access among EU citizens [6, 27, 40]	EU countries	‘*Levelling of prices signifying less affordable pharmaceutical products’*	There is no evidence on whether ERP in itself had an effect on social welfare; in combination with parallel trade, the use of ERP was associated with an effect on social welfare
	ERP impacts health system priorities	ERP might be primarily relying on pricing factors extrinsic to the health care system in which it operates and subsequently might neglect country-specific health system priorities [2, 3, 8, 46].	N/A	N/A	
Microeconomic efficiency	ERP has an impact on efficient drug spending (through price revision)	ERP may potentially increase efficiency in terms of affordable prices, especially through frequent periodic price revisions of listed drugs [5]	Slovakia, Switzerland	Slovakia: ERP based on the arithmetic mean of the six lowest countries within EU 26 countries, resulted in a 25% reduction in pharmaceutical expenditure as proportion of total health care spending	Examples from the literature highlighted the short-term impact of ERP on efficient drug expenditure by lowering prices, although no conclusive evidence was found to assess whether the impact of ERP on social equity and welfare is short- or long-term [5]
	Contributes to stable share of drug spend as proportion of total health spend	ERP might have the ability to reduce the share of pharmaceutical expenditure in total health care spending [5]	Slovakia, Switzerland	Slovakia: ERP based on the arithmetic mean of the six lowest countries within EU 26 countries, resulted in a 25% reduction in pharmaceutical expenditure as proportion of total health care spending	
	Containing costs while guaranteeing access to medicines	Evidence on the impact of ERP on efficiency in the context of cost-containment, while maximising accessibility is inconclusive [6]	N/A	N/A	
Industrial policy	ERP impacts innovation and investment in R&D	ERP may discourage incremental innovation and investment in R&D through: (a) downward price convergence potentially leading to reduced revenues for pharmaceutical companies, (b) encouragement of parallel trade potentially leading to manufacturers’ investing in producing only marginal product modifications in order to avert the threat of parallel trade and (c) the ERP objectives or rules themselves, and the way they are implemented in different settings and which do not necessarily support industrial policy [3, 6, 15, 49, 51]	Slovakia, Germany, Hungary, Egypt, Spain, Latvia, The Russian Federation, South Africa, Qatar, Greece, Romania, Italy, Estonia	Poorly defined ERP rules in Germany render firms less able to profit from incremental innovation in drug discovery	Very limited evidence suggests that ERP might deter manufacturers from investing in R&D in the short-term [23]
	ERP influences manufacturing and/or R&D investment decisions	There is no clear evidence on the impact of pharmaceutical policy practices on industrial policy decisions in Europe due to the multiplicity of factors involved and the long causality chain linking non-regulated pricing to innovation [48]	N/A	N/A	
	ERP offers scope for promoting innovation	ERP could indirectly incentivise innovation through favourable basket and other parameter definition. Innovation may be rewarded in the context of defining the basket of comparators (i.e. inclusion of countries that explicitly recognize value and the “value of innovation”) or in the context of adjusting prices frequently to reflect price adjustments in other settings [8]	Slovakia, Belgium, Brazil, Jordan	N/A	

*N/A* not applicable, *R&D* research and development, *EU* European Union, *ERP* external reference pricing, *GDP* gross domestic product, *m* million, *bn* billion, *OECD* Organisation for Economic Co-operation and Development, *LICs* Low Income Countries, *PPP* Purchasing Power Parity, *POM* prescription-only medicine

### ERP and impact on cost-containment

Where implemented for price-setting purposes and depending on its design, ERP is expected to have a downward effect on prices and contain pharmaceutical expenditure; the latter can be interpreted as either reducing pharmaceutical costs or containing their rate of growth. However, lower prices do not necessarily translate to overall pharmaceutical expenditure reductions; these also depend on other factors including drug utilisation. Consequently, the extent to which ERP has the ability to reduce pharmaceutical expenditure or contain its rate of increase is unclear unless key important confounders are explicitly accounted for. In this section we discuss the evidence on the impact of ERP on the overall healthcare and pharmaceutical spending, while the impact on price levels and drug use is discussed separately later in the relevant section.

Evidence suggests that in Europe “the conditions on the EU market are in effect weakening the use of cost-based price regulation and giving more importance to the observed price in other European countries using external reference pricing” [16]. Across Europe, ERP has sometimes been shown to be effective in generating considerable savings for public payers in the short-term, largely depending on the ERP methodology applied, while, ERP impact on healthcare savings in the long-term is highly dependent on the pricing policies and the economic conditions existing within the country and across reference countries. The limited ability of ERP to serve as a cost-containment tool in the long-term is partly attributable to the ‘fadeout’ effect, where ERP was successful in the short-term but has gradually lost its effectiveness [1]. This effect has also been observed with other pricing and reimbursement tools beyond ERP, where further adjustments were made in their implementation to regain their degree of effectiveness [1].

In Slovakia, the new reference system was expected to create savings estimated at €75 million by the end of 2012 due to price reductions caused by ERP, but no evidence exists as to whether this target was actually fulfilled. This reformed ERP system set pharmaceutical prices based on the average of the two lowest in the EU, replacing the previous system, in which prices could not exceed the average price of the six lowest EU prices [17]. In a simple pre-post study without adjustment for confounders, it was found that in Turkey in 2007, ERP led to considerable reductions in pharmaceutical prices, leading to savings for the government of about US$ 1 billion [18]. By contrast, when ERP was implemented in Greece in 1996 it initially led to a reduction in public pharmaceutical spending but proved to be ineffective over the long-term as pharmaceutical expenditure continued to rise at rates similar to those before its introduction. This can be attributed to the replacement of older products by new products within the same therapeutic category that were more expensive and more widely prescribed by physicians. It has been concluded that, at least in Greece, emphasis on price controls only is not effective in containing pharmaceutical expenditure if it is not accompanied by any policy interventions to control proxy demand (prescribing and/or dispensing) and overall volume consumed [19, 20].

While savings are likely to occur for publicly funded health care systems, the extent of such savings depends largely on the way ERP is implemented. In Switzerland, in 2010 and 2011, changes in the implementation of the ERP system were made putting downward pressure on prices [21], by increasing the number of basket countries used as reference and initiating more frequent price revisions [12]. Additionally, in a series of simulation exercises, healthcare savings were found to be higher when net or discounted (rather than list) prices are used compared with regular price revisions [1]: a discount or rebate of 20% on pharmaceutical prices in large markets with high GDP per capita (Germany, France, the UK, Italy, Spain, the Netherlands and Switzerland) would deliver an average pharmaceutical price decline of 47% across EU countries implementing ERP; this is attributable to the above countries being used routinely in the reference basket of most other European countries. Therefore, referencing net or discounted prices instead of list prices will directly affect prices in all European countries using ERP by lowering basket averages [1]. In comparison, more frequent price revisions (e.g. every 6 months) over a period of 10 years would result in a reduction of about 6% in pharmaceutical prices in European countries implementing ERP. As frequent re-evaluations can be administratively burdensome, conducting them should be balanced against the expected benefit [1].

### ERP and impact on price levels

Extensive evidence from the literature highlighted that when ERP is first introduced in a country it reduces pharmaceutical prices at least in the short-term [4, 5, 8, 16, 22]. However, the long-term effects are unknown and, indeed, primary evidence on the ability of ERP to lead to, and secure, low prices on a sustainable basis, is mixed. It is important to highlight that evidence derived from both the literature and the survey does not distinguish between nominal and real prices.

The introduction or reform of ERP seemed to be associated with price reductions in a variety of settings, including the Netherlands (pharmaceutical average price level declining by 8% between 2007 and 2008) [23], Cyprus (based on a simulation exercise testing the effects of a likely ERP introduction, after identifying Cyprus as a “high-price” country for pharmaceuticals) [24], Norway (where it has been regarded as very successful since 2009, resulting in considerable and predictable price reductions) [25], Moldova (where the 2010 reform of ERP, setting ex-manufacturer prices equal to the average price of the three lowest prices in the basket, lowered prices by 3% in 2011) [26], Romania (where the prices of prescription pharmaceuticals were found to be low compared to the EU average due to the use of ERP in 2014) [27], Bulgaria (where the government changed the ERP design in 2012, such that the basket was increased from 8 to 12 countries and yearly price checks were implemented for all reimbursable pharmaceuticals, leading to price reductions for reimbursed pharmaceuticals by between 4 and 75.4%) [22, 28–30] and Greece (where changes to the ERP system in September 2010 resulted in an average price decrease of 9.5% compared with the prices attained from the temporary price cuts regulation in May 2010) [31]. Although the evidence derived from both the literature and the survey does not distinguish between nominal and real prices, in practice, all studies refer to current or nominal prices.

Pharmaceutical price levels within a country are influenced predominantly by the nature, rules and stylised features of the implemented ERP system. Upon implementation of ERP, both primary and secondary evidence suggests that the most influential parameters on the evolution of pharmaceutical prices over time are the frequency of price revisions, the size of the country basket, the ERP formula used to determine list prices and exchange rate fluctuations [4, 7, 8, 15, 32–36]. In various simulation exercises analysing the reaction of pharmaceutical prices to different ERP modalities, ERP systems led to reductions in pharmaceutical prices when (a) frequent price revisions and iterative price cuts were applied based on these revisions, (b) country baskets were very large, (c) a country used the lowest price or average of the three lowest prices in their basket rather than the average or median price when calculating list prices, and (d) exchange rate fluctuations were used proactively to achieve price reductions in local currency [7, 8].

Countries with no price revisions over time did tend to have limited price changes. In a simulation exercise testing drug price evolution of 13 countries over 10 years, countries with the smallest price decreases were those that had implemented no or less frequent price revisions (Austria, Belgium, Cyprus, Denmark, Estonia, Germany, Hungary, Iceland, Luxembourg, and Poland), while the highest decreases were observed in countries with frequent price revisions (Greece, Latvia, Lithuania and Slovakia) [7]. Primary evidence from Hungary confirmed that price decreases in the countries of its basket could not be reflected in the Hungarian reimbursement system because ERP would be used at launch only [15].

The effect of local currency depreciation on prices is also demonstrated in the case of the Russian Federation, where primary evidence indicated that the prices of internationally manufactured pharmaceuticals that were first registered in 2009 had not undergone any price revisions until 2016, despite significant devaluation of the rouble. In 2016, the Federal Antimonopoly Service (FAS) of the Russian Federation initiated price revisions and revised prices declined compared to  those in 2009 [15].

Changes in the mix of basket countries have also been shown to influence price levels downwards. A modification in the Croatian ERP system in 2012 through which France was removed from its reference basket and replaced by the Czech Republic led to a reduction in most pharmaceutical prices [35]. Primary evidence from Spain indicated that price levels would depend predominately on the countries used as reference: over time, it was observed that prices would increase when countries with higher GDP per capita than Spain were included in the Spanish reference basket; in a similar vein, prices would decrease when countries with lower GDP per capita were used in the basket [15]. A similar trend was observed in Slovakia, where ERP initially tended to result in higher prices relative to neighbouring countries with similar income levels due to reference country selection, particularly because Germany and originator country prices were used to calculate reference prices; a policy change using the mean of the six lowest countries in Europe was implemented in 2009 leading to lower prices [4, 36]. The experience of Switzerland is in the same direction as the increase in the number of countries in its basket in 2010 led to a higher possibility of further pharmaceutical price reductions [21, 32–34]. Lithuania expected that the inclusion of two low price countries (Bulgaria and Romania) in its basket in 2012 would exert downward pressure on prices [37, 38]. Finally, an EU study analysing the effect of ERP on price levels in seven European countries for 11 pharmaceutical products between January 2003 and December 2008 found price reductions in four of the seven countries, all of which calculated reference prices using the average of the *n* lowest of the basket, or the lowest available price in the basket [8].

Other market-specific aspects can influence the impact of ERP on list price levels, such as the country income level, the health needs of the population and healthcare costs [4–6, 15]. However, because confidential rebate data are not available, it is unclear what impact such data might have on actual price levels and, indeed, on reimbursed prices. An empirical study comparing list prices in 14 European countries and using multiple regression analysis concluded that prices were generally lower when a country applies ERP, compared with other systems of pharmaceutical pricing, including non-price interventions. Primary evidence from Qatar suggested that the introduction of ERP led to lower prices, following substantial price increases attributable to a free pricing system [15]. Further empirical work estimated that countries with stricter price regulation (Portugal, Belgium, Greece, Spain, as well as France and Italy, at least in earlier years) experience lower prices than less regulated markets. However, the former face longer launch delays and access issues may ensue [39]. Finally, GDP per capita and price levels seem to be positively related: countries with a high GDP per capita (e.g. Norway, the Netherlands, Finland, Austria and Belgium) have higher price levels than countries with lower GDP per capita (e.g. Spain, Greece and Portugal) [4].

ERP may hinder generic penetration or even drive an in-patent originator out of a market creating availability problems in specific circumstances where low generic prices are available in certain reference countries and are taken as benchmarks by ERP systems elsewhere. This can generate unsustainably low price levels—particularly where tendering practices are implemented—as well as eliminate patent rights if patent expiries differ between reference countries and ERP implementation countries [6].

While there seems to be some demonstrable effect on price reductions, ERP has been criticised for not having a noticeable impact on price levels [8] and has been characterised as a “non-optimal” pricing regulation for ensuring appropriate and competitive price levels compared with other pharmaceutical pricing systems, because (a) it impedes price flexibility within individual settings and (b) tends to reinforce narrow price corridors across settings [40]. Importantly, prices subject to ERP are increasingly disconnected from transaction prices. As transaction prices are not publicly available, countries implementing ERP adopt increasingly fictitious list prices, which tend to be systematically and substantially higher than transaction prices, thereby artificially boosting list price levels [3, 6, 8, 12]. Survey responses from Estonia and Latvia question whether ERP leads to lower or higher prices as reference prices do not reflect transaction prices and any additional confidential price discounts agreed; confidentiality restrictions, rebates, discounts, clawbacks and, in general, any price negotiation between third party payers and companies, lack transparency and cannot be considered under ERP [15]. Both primary and secondary evidence showed that the impact of ERP is limited in lowering pharmaceutical prices within countries because it is unable to account for the lower discounted prices when referencing other countries [2, 4, 5, 8, 15].

The availability of list (rather than net) prices suggests that countries using ERP may reference artificially high prices, while in the long-run this phenomenon renders ERP ineffective and irrelevant as confidential discounting and rebating are applied widely in pharmaceutical prices [3, 12]. On the other hand, official list prices offer flexibility around negotiations taking place between manufacturers and pricing authorities [40].

Overall, 11 of the 26 studies studying, among others, the relationship between ERP and prices used quantitative data to study the impact of ERP on pharmaceutical prices. The majority of studies presenting evidence on the impact of ERP on price levels were descriptive using data collected via post-only designs.

### ERP and impact on drug use

Evidence on the impact of ERP on controlling drug consumption within a country comes only from the stakeholder survey [15]. This is hardly surprising as consumption is influenced by a variety of other parameters, such as the number of prescribing doctors, the incentives driving prescribing behaviour and patient demand, among others. Primary data from Brazil, Egypt, France, Greece, Hungary, Latvia and Qatar, suggest that the uptake and the use of pharmaceuticals are not affected by the ERP system in place, indicating that product diffusion is not hindered once the pricing decision has been made [15]. However, primary data from Hungary and Greece indicated that if the budget impact of a product is thought to be high, the patient population having access to that product can be restricted in relation to its marketing authorisation indication [15]. Insights from Slovakia, Spain, Bulgaria, Estonia, the Russian Federation and Jordan, indicated that ERP systems were considered a necessary but not sufficient condition for the optimal diffusion and use of pharmaceuticals [15]. When ERP was implemented in Italy in the past, it was thought to be associated with reduced diffusion and availability of pharmaceuticals [33]. Lower prices resulting from the version of ERP system implemented in Italy encouraged parallel exporters to shift products from the Italian market to high-price countries, thus reducing local availability and, by implication, adversely affecting product diffusion [15].

### ERP and impact on availability and launch delays

ERP may indirectly hinder the availability of medicines [2, 41, 42] in a number of ways, including product withdrawal or discontinuation, launch delays, launch sequencing and parallel trade. As list prices are usually set based on the average of the lowest or the lowest price in a country's basket, it may result in a general price decrease when one country from the basket reduces its price. To avoid lower prices cascading from one country to another, manufacturers have a number of options: for those products already on the market, manufacturers can withdraw from the market. For new products awaiting launch, ERP can lead to launch delays, launch sequencing, or no launch, resulting in non-availability even on an out-of-pocket basis, as manufacturers do not achieve desired price levels [2, 28].

Several literature sources have assumed that ERP might lead to product shortages in countries referencing the lowest price, due to discontinuations and parallel exports [3, 6]. This is also supported by primary evidence, particularly from European countries [15], where ERP is widely practiced and where the principle of regional exhaustion of intellectual property rights applies, which is a necessary condition for parallel trade to take place. A study on the short- and long-term effects of ERP in Europe found a discernible impact on availability in the countries included in the analysis, where manufacturers did not launch several products to avoid having to launch products at low prices [8]. Others have also specifically linked non-availability of medicines to the concept of “launch sequencing” due to ERP, whereby companies delay or withhold product launches in countries with highly controlled prices because of the knock-on effect lower prices will have elsewhere [1, 5–8, 36]. Overall, due to ERP policies, fewer drug launches and longer drug launch periods are likely to be the case in highly regulated and/or small markets compared to markets with relative flexibility on pricing, or markets that are large in size, with higher GDP, a higher percentage of GDP devoted to health and a higher price level of pharmaceuticals [3, 25].

Respondent insights from Slovakia, Hungary and Romania, indicated that availability issues are caused by national pricing regulations [15]. By contrast, both prices and availability are highest where prices are not regulated. An example of launch sequencing strategies due to ERP was Belgium, where companies systematically delayed dossier submission to avoid Belgian prices being included in other countries’ price-setting processes [7]. In recent years, however, Belgium has transitioned to a system of value assessment and no longer relies on ERP; as such, no availability issues were documented since that transition [15].

In Slovakia, a change in its reference basket to include all EU Member States resulted in companies disregarding the newly implemented prices or lobbying for exemptions to their products, leading to access delays [5]. Similarly, in Bulgaria, around 200 products were withdrawn from the market in 2012 due to the very low prices attained by ERP [6, 7]. The same was mentioned in Spain few years ago, however, currently, due to the dual pricing system, no availability problems are documented [15]. Primary data from Estonia showed that there were product withdrawals when manufacturers were not willing to lower pharmaceutical prices and when reasonable therapeutic alternatives were present on the market, while in Romania, Slovakia and Poland instances of product withdrawals were also observed [15], suggesting that even if patients would be willing to pay out-of-pocket, withdrawn products would not be available. While some studies argue that ERP poses significant threat to the accessibility of medicines, particularly if any of the reference countries implement strict pharmaceutical expenditure policies due to the economic crisis [43], other studies explicitly recognise that their results should be interpreted with caution [3, 8]. Overall, if products are not reimbursed by a country’s health care system, in principle, they might be available to purchase on an out of pocket basis; it is not uncommon, however, that unless a (reimbursement) price has been agreed and which will apply to the entire market, the product may not available at all.

### ERP and impact on affordability

Affordability is the extent to which pharmaceutical prices in a country align to the purchasing ability of the healthcare system and/or of patients. Affordability concerns have been raised by several survey experts in Slovakia, Belgium, Brazil, Bulgaria, Spain, France, Greece, Hungary, Italy, Russia, South Africa and Jordan and for some therapeutic categories [15]. In Egypt, literature evidence, confirmed by expert opinion, showed that actual list prices of in-patent products, based on an extensive ERP basket of 36 countries, declined by a further 10% following ministerial requirements to improve local affordability. As some low- or middle-income country baskets include high income (and, potentially, high price) countries, this could potentially trigger issues with affordability, unless policy makers change pricing policies to adapt to the new market dynamics [44], for example, by considering a kind of affordability index, whereby pharmaceutical prices are weighed by GDP at exchange rates or purchasing power parities (PPPs) [1, 2]. In Egypt reforms have been put on hold to allow authorities to consider a better alignment between Egypt and reference counties in terms of PPPs, although the impact of the pricing reform will remain unknown until the authorities review the PPP in relation to the potential reference countries [45]. To achieve affordable access to medicines, middle income countries may need to rely on confidential agreements with manufacturers to obtain lower prices through rebates or discounts and further discourage any external spillover impact of their list prices [44]. Contrary to the evidence presented above, primary evidence from Qatar, Latvia and Estonia showed no detectable issues with affordability [15].

### ERP and fairness/social welfare

Hardly any evidence was available studying welfare effects of ERP directly other than studies investigating price, use, availability or affordability issues, thereby arriving at social welfare impact indirectly. Much of the evidence on this endpoint comes from an evaluation of international dimensions of ERP and, specifically, parallel trade. Within the EU context, it has been shown that ERP's tendency to downward price convergence and the opportunity to conduct parallel trade between low- and high-price countries, (a) impacted social welfare by causing spillover effects from low- to high-price countries, (b) led to barriers in patient access in low-price markets and (c) delivered limited benefits to health systems and patients in terms of cost savings in high-price markets [6]. Therefore, ERP may undermine equitable and affordable patient access, particularly in low-price, low-income countries [27]. Research also concluded that ERP might be primarily relying on pricing factors extrinsic to the health care system in which it operates [8] (by importing prices from other settings) and, as a result, may not reflect or address country-specific health system priorities, for instance by calibrating prices of new products based on current system needs. For instance, this phenomenon was observed in the Russian Federation, where the national priority was to increase savings on the health care budget [46].

Several examples have highlighted that, often, countries pursue “beggar-thy-neighbour” practices to capitalise on the possibility of low prices; for example, Belgium and Austria (with a GDP per capita of US$37,883 and US$42,408, respectively in 2012) referencing Romania (per capita GDP of US$12,802) and Bulgaria (US$14,301) or Ukraine (US$7,374) referencing Moldova (US$3,415), and Pakistan (US$2,880) referencing Bangladesh (US$2,093). Although there is no indication that price levels in referenced countries are (much) lower than in referrer countries, the latter seem to want to capitalise on any price differences irrespective of country archetype or per capita income level. In principle, such practices nurture inequalities among countries, as wealth differences between referrer and referenced country proliferate [3].

### ERP and impact on macroeconomic efficiency

Evidence on the impact of ERP on the efficiency of the health care system and its ability to lead to effective resource allocation is very limited. A descriptive overview of national ERP systems in EU countries showed that, in terms of efficiency, ERP led to a 25% reduction in the proportion of pharmaceutical expenditure as a percentage of total health care spending in Slovakia, when the country adopted the EURO in 2009. This was accompanied by a change in its ERP policy, which introduced the arithmetic mean of the six lowest-priced countries across EU Member States [5]. A Swiss study argued that reliance on external and internal price benchmarking as a basis for price setting, rather than pharmacoeconomic assessment, could help  'optimise' a country’s pharmaceutical expenditure [47]; in doing so, the potential of ERP as a mechanism to enhance efficiency in pharmaceutical expenditure was recognised, particularly through frequent periodic list price revisions, although assessment of the impact of these revisions was not available. Finally, one source assessed the impact of ERP on macroeconomic efficiency in the context of cost-containment while maximising accessibility, but the evidence was inconclusive [6].

### ERP and impact on industrial policy

It has been argued that price convergence, generated by ERP-based systems, discourages innovation by reducing revenues and acting as a disincentive for continued research and development (R&D) investment [6], yet the evidence is not clear due to the multiplicity of factors involved and the long causality chain linking factors such as non-regulated pricing to innovation [48]. Discouraging innovation could be due to countries using various determinants in their ERP setup, but not clearly explaining whether and how these determinants are valued or combined, creating regulatory uncertainties, which might ultimately discourage manufacturers from investing in R&D [3]. Survey evidence from Egypt, Spain, Latvia, Russia, South Africa, Greece, Hungary, Romania, Italy, Estonia and Qatar suggested that the objectives of local ERP systems do not promote industrial policy [15]. Under these broad rules, research-based manufacturers will find themselves less able to profit from incremental innovation [49–51]. Despite the above observations, it has been suggested that even though encouragement of and reward for innovation are not explicit objectives of ERP itself as a policy tool, a positive attitude towards innovation may be achieved either by calibrating reference baskets to include countries that explicitly implement value assessment methods, or even by implementing ERP as a ‘light’ option, for example, at launch only [8].

### Quality of evidence

Evidence on the impact of ERP on healthcare savings was limited and descriptive in nature. Half of the studies identified on the impact of ERP on price levels were quantitative, thus containing empirical evidence on impact (last column, Table 2), while the other half contained statements on impact rather than hard evidence captured in monetary terms or percentages. There was no evidence from the literature on the impact of ERP on drug consumption. The evidence on the quantifiable impact of ERP on market withdrawal and/or launch delays of pharmaceutical products was of very low quality (Table 3). Although some empirical evidence was identified regarding the impact of ERP on affordability of medicines within a country, little was identified in terms of quantifying the extent to which affordability of medicines would be affected as a result of ERP policies. A very limited body of evidence, largely descriptive in nature, was identified on the impact of ERP on equity, while literature relevant to the impact of ERP on macroeconomic efficiency was scarce and inconclusive. Finally, the available empirical evidence on the effects of ERP on industrial policy was both scarce and of very low quality. Table 3 presents the overall direction (positive vs. negative vs. inconclusive) and quality of evidence found in the literature on the impact of ERP at country level.

**Table 3:** Overall direction and quality of evidence from the literature on the impact of ERP at national level. Source: Synthesis and assessment by the authors based on primary and secondary data collection

Study endpoints	Issues identified within endpoints on ERP impact	Impact of ERP positive (+) negative (−) or ambiguous (±)^a^	Quality of empirical evidence on the impact of ERP (where applicable)^c^	Duration evidence applies to: short-term (S) or long-term (L)^d^
Cost-containment	Generating healthcare savings	**+**	Very low	S
	Magnitude of healthcare savings depends on ERP design	**±**	Very low	
Prices/price levels	Achieves lower pharmaceutical prices	**±**	Very low	S/L
	Pharmaceutical prices depend on ERP design	**±**	Very low	
	Pharmaceutical prices depend on market features	**±**	Very low	
Drug use	ERP helps contain consumption	**±**	Not Available	S
	ERP improves drug diffusion and use	**±**	Not Available	
Availability and launch delays	Possibility of withdrawal from market	**+**	Very low	S/L
	ERP causes launch delays, launch sequencing or no launch	**+**	Very low	
Affordability	ERP leads to pharmaceutical prices in line with the purchasing ability of healthcare systems or patients	**±**	Very low	S
	ERP provides scope for increasing affordability	**±**	Very low	
Fairness/social welfare	ERP can lead to social welfare improvement	**-**^b^	Not Available	S
	ERP may neglect country-specific health system priorities	**−**	Very low	
Microeconomic efficiency	More affordable prices through price revision	**+**^b^	Not Available	S
	Contributes to stable share of pharmaceutical expenditure on total health spend	**±**	Not Available	
	Contains costs while guaranteeing access to medicines	**±**^b^	Very low	
Industrial policy and innovation	May discourage incremental innovation and investment in (incremental) R&D	**−**^b^	Very low	S
	May influence manufacturing and/or R&D investment decisions	**+**^b^	Very low	
	May indirectly incentivise innovation	**±**^b^	Very low	
Overall		**±**	Very low	S

^a^Regarding the direction of impact of ERP, the “+” sign indicates that ERP contributes to achieving the stated goal(s); the “−” sign indicates that it does not contribute to achieving the stated goals. The sign “±” is used in those cases where the impact of ERP on the relevant endpoint and issue is ambiguous. This is generally observed when the impact of ERP depends on other factors, such as the modalities of ERP methodology or other exogenous factors. In order to arrive at the direction of impact as shown, a simple-vote counting methodology was adopted by counting the number of identified studies providing positive evidence and the number of those providing negative evidence

^b^Inconclusive evidence

^c^The overall quality of the identified empirical evidence has been classified as “high”, “moderate”, “low”, “very low” and not available. During vote counting only studies examining each endpoint/issue empirically were considered for quality assessment. As discussed in the Methods section, some studies referencing evidence using a post-only design were classified as “very low” quality, whereas studies performing regression analysis were considered to be of “low” quality. Where quasi-experimental designs or difference-in-difference methodologies were used, the quality of evidence was classified as “high”. Under each endpoint/issue, when different types of empirical studies were considered, the quality of evidence was assessed based on the majority, for example, when empirical evidence under an endpoint was given by three studies using a post-only design and one study using a regression analysis design, then the quality of empirical evidence under this endpoint was considered as “very low”

^d^The last column describes the duration of the relevant evidence and indeed whether the evidence provided under each endpoint/issue considered the short or long-term impact of ERP, denoted by “S” or “L”; “S/L” denotes circumstances where both short- and long-term impact are considered

## Discussion and policy implications

As evidence found in the literature on the impact of ERP on a number of health system objectives was weak and of low quality in the majority of cases, primary data were collected to validate, complement, update and enhance our understanding of the impact of ERP at country level. Overall, ERP seems to contribute to cost containment by exerting downward pressure on pharmaceutical prices, at least in the short-term following its introduction. However, the magnitude of healthcare savings depends largely on ERP design. Increases in the number of basket countries as well as more frequent price revisions are likely to lead to greater reductions in pharmaceutical prices and will in principle generate healthcare savings. Nevertheless, these savings can be limited because transaction prices remain elusive and list prices, which are used in reference price setting, are increasingly fictitious.

Results on the impact of ERP on price levels varied between secondary and primary evidence. Based on the literature, upon its introduction, ERP has the ability to reduce pharmaceutical prices, whereas mixed evidence was documented from primary sources on this endpoint. This is because price levels within countries are influenced by the nature and the rules of the ERP system. Again, frequent price revisions, the basket size and the formula used to calculate reference prices can determine the extent of price reductions observed within countries. A study in the same series discusses how ERP is implemented and the variations observed in the ERP design across countries [9]. Other aspects of the market can have an impact on price levels, such as country income level, the health needs of the population and overall healthcare costs. Despite the documented price declines, ERP has been criticised both in the literature and by experts as being a “non-optimal” pricing regulation not resulting in appropriate and competitive price levels over time compared with other approaches to pharmaceutical pricing, such as cost-effectiveness or value-based pricing (VBP). When a country refers to source settings where VBP is applied, additional concerns may arise about the ability of ERP to secure low prices where it is applied: as reimbursement is often available for selected (usually high-risk) populations, prices in source countries can be higher than might otherwise be the case [52].

Over time and in a number of settings, the role of ERP in price setting has transitioned from primary to supplementary. To that end, ERP is either the starting point for price negotiations or is used in combination with other tools to determine prices; this highlights the limits of ERP’s effectiveness as a pricing tool, since it is based on list rather than net prices. Consequently, ERP-derived prices are not considered final, but, rather, an upper ceiling for the purposes of negotiation [9].

The weak link between ERP and drug use was highlighted in the survey where the importance of arrangements and incentives on the proxy demand- (through approaches to prescribing, dispensing, clinical guidance and financial and non-financial incentives) and the demand-side (cost-sharing), were underlined.

Downward price convergence and the prospect of reduced manufacturer revenues can be detrimental for medicines availability and incentivize manufacturers to adopt launch sequencing strategies, in an attempt to avoid a downward price spiral by delaying the launch of new products in low-price countries or in countries with highly regulated prices at ex-factory level. Again, the modality of ERP implementation is important and seems to partly determine the extent of launch sequencing and, through that, availability. For example, ERP at launch only does not seem to have an impact on availability, whereas ERP associated with frequent price revisions and combined with unrealistic exchange rates or intense exchange rate fluctuations, does.

Availability problems may also be triggered by different patent expiries between jurisdictions. Circumstances have been reported where patent expiry and subsequent generic entry at a significantly reduced price in one setting has triggered price revisions in another where the drug in question is still on-patent; if the price diminution in the latter is based on the generic price borrowed from the former, there is a risk of withdrawal from the market if an on-patent originator is assigned the price of the generic. In this case, while the objective of decision makers is to improve affordability, the end result may be reduced availability, as the on-patent product may be withdrawn from the market if exposed to imported generic price competition. The broader implication is that, if this occurs on a frequent basis and across settings, it will unavoidably carry negative implications for long-term commitments to R&D investment apart from the issues related to availability.

As far as affordability is concerned, several countries rely mostly on confidential agreements to obtain lower net prices through rebates and discounts. ERP does not seem to promote efficient resource allocation and may shift the welfare equilibrium within a country due to higher, unaffordable prices relative to the GDP per capita of the country. Undoubtedly, there is a bi-directional relationship between the impact of ERP within a country and across countries. Launch delays in third countries could result in non-availability of pharmaceuticals in some small and low-income countries. Launch-sequencing strategies adopted by manufacturers can lead to reduced availability of medicines in smaller markets or in countries with lower prices. Therefore, when studying the impact ERP has on a country, the impact ERP has beyond a country’s borders needs to be acknowledged [53].

Despite the relative abundance of studies found on the impact of ERP on cost containment, prices, availability and launch delays compared to other endpoints, evidence on the quantifiable impact of ERP on these endpoints is of low quality and largely inconclusive to support claims about sustainable savings, price diminution or poor availability due to ERP. For example, the validity of evidence provided on the quantifiable impact of ERP on healthcare savings is limited and subject to criticism as the majority of studies were descriptive, using a post-only design; only three studies assessed the ability of ERP to generate healthcare savings over time [1, 19, 20]. Overall, therefore, the strength of evidence regarding cost containment was found to be weak and the quality of evidence low. Some evidence was found in relation to the impact of ERP on affordability of medicines within a country, but little of substance was identified that would quantify the extent to which affordability of medicines would be affected as a result of ERP policies. A very limited body of evidence, largely descriptive in nature, suggests that ERP does not necessarily reflect the goals and priorities of the health system in which it operates, that it may shift the welfare equilibrium within a country due to higher pricing and that subsequent affordability issues arise as a direct consequence of ERP. Equally, literature on the impact of ERP on macroeconomic efficiency is limited and inconclusive. Consequently, the quality of evidence on the impact of ERP within a country is weak, it emerged from a limited number of empirical studies, the majority of which were based on qualitative analyses of survey data or regression analyses of observational data, which could not be controlled for bias and/or potential confounders. No relevant studies were found that assessed or quantified the impact of ERP by employing methods that allow for causal inference, such as time series or panel data analyses. Based on this, it is difficult to draw distinctly positive or negative conclusions about the effects of ERP. As such, some of the results of this review should be interpreted with caution and care should be exercised when inferences are made about the long-term implications of ERP.

Our analysis is not without limitations. The first limitation relates to our results being limited to the English language only and there may be studies in other languages, which we could not account for. However, the expert input into this study will have mitigated the extent of gaps in our analysis. A second limitation relates to a substantial proportion of studies reviewed being quite dated; this is important as countries may have moved to alternative methods of pricing. Similarly, expert insights have been useful and provided updated evidence. Third, the effect of ERP as an isolated policy is very difficult to disentangle in the presence of other regulatory interventions implemented both within the country that implements it, but also in reference countries. Therefore, in the presence of several confounders, it is not possible to draw robust conclusions on the impact of ERP. Fourth, data on discounts and rebates remain confidential, therefore our study of the impact of ERP is limited to list prices rather than transaction prices. Given the confidentiality surrounding transaction prices, it is doubtful that this can be addressed in any particular way. Finally, as pharmaceutical pricing policies are constantly undergoing updates and reforms, the evidence presented in this study may not reflect the policy landscape in future years. However, this study provides a benchmark at this point in time for future analyses.

## Conclusions

Based on a combination of primary and secondary evidence on the impact of ERP within countries, the following conclusions can be supported at national level: first, when ERP is implemented, pharmaceutical expenditure can be contained or even decline at least in the short-term because prices may decline; however, this effect may altogether be annulled if there is no effective control on volume. Second, the availability of and equitable access to pharmaceuticals, as well as the promotion of industrial policy can be undermined when ERP is used to inform pricing at national level. Third, the impact of ERP on the affordability of medicines is ambiguous and subject to the design features of the policy. Fourth, the impact of ERP on policy objectives such as sustainable price levels, cost containment and availability, depends predominantly on the way ERP is implemented in a specific setting as well as on extrinsic factors, such as launch delays in other countries. Finally, this is the first review that critically assesses the quality of the evidence found in the literature on the impact of ERP on health and pharmaceutical policy objectives. We have identified studies, the majority of which rely on weak non-experimental study designs and conduct post-only analysis. To some extent, therefore, the results identified above need to be interpreted with caution as it is not possible to make inferences about the impact of ERP on individual policy variables and its overall impact within countries. Longer-term studies and better designs are needed in this context to draw more robust conclusions. Overall, it has been shown that ERP constitutes one approach to pricing; however, it is implemented with a variety of different modalities depending on the salient features used each time. Whereas some of the ERP implementation modalities may be associated with negative implications regarding access, availability or delays in product launches, other modalities are more neutral to these critical variables. Despite its obvious limitations, ERP should not be altogether dismissed as improvements can clearly be made in the way it is implemented.
